# NOTCH4 mutation as predictive biomarker for immunotherapy benefits in NRAS wildtype melanoma

**DOI:** 10.3389/fimmu.2022.894110

**Published:** 2022-07-29

**Authors:** Hongxia Li, Qin Zhang, Qianqian Duan, Yuan Tan, Tingting Sun, Chuang Qi

**Affiliations:** ^1^ Department of Oncology, Shanxi Provincial People’s Hospital, Taiyuan, China; ^2^ The Medical Department, Jiangsu Simcere Diagnostics Co., Ltd., Nanjing, China; ^3^ The Medical Department, Nanjing Simcere Medical Laboratory Science Co., Ltd, Nanjing, China; ^4^ The State Key Lab of Translational Medicine and Innovative Drug Development, Jiangsu Simcere Diagnostics Co., Ltd., Nanjing, China

**Keywords:** melanoma, NRAS wildtype, immune checkpoint inhibitors, biomarker, anti-tumor immunity

## Abstract

**Background:**

NRAS wildtype melanoma accounts for approximately 80% of melanomas. Previous studies have shown that NRAS wildtype melanoma had higher response rates and better prognoses than NRAS-mutant patients following immunotherapy, while as major actors in tumor cells and tumor microenvironment (TME), the association between NOTCH family genes and response to immunotherapy in NRAS wildtype melanoma remains indistinct.

**Objective:**

We aim to explore whether NOTCH family gene variation is associated with genomic factors in immune checkpoint inhibitor (ICI) response in NRAS wildtype melanoma and with clinical results in these patients.

**Method:**

This research used genomic data of 265 NRAS wildtype ICI-pretreatment samples from five ICI-treated melanoma cohorts to analyze the relationship between NOTCH family gene mutation and the efficacy of ICI therapy.

**Results:**

NRAS wildtype melanomas with NOTCH4-Mut were identified to be associated with prolonged overall survival (OS) in both the discovery (HR: 0.30, 95% CI: 0.11–0.83, *P* = 0.01) and validation cohorts(HR: 0.21, 95% CI: 0.07–0.68, *P* = 0.003). Moreover, NOTCH4-Mut melanoma had a superior clinical response in the discovery cohort (ORR, 40.0% vs 13.11%, *P* = 0.057) and validation cohort (ORR, 68.75% vs 30.07%, *P* = 0.004). Further exploration found that NOTCH4-Mut tumors had higher tumor mutation burden (TMB) and tumor neoantigen burden (TNB) (*P <*0.05). NOTCH4-Mut tumors had a significantly increased mutation in the DNA damage response (DDR) pathway. Gene set enrichment analysis revealed NOTCH4-Mut tumor enhanced anti-tumor immunity.

**Conclusion:**

NOTCH4 mutation may promote tumor immunity and serve as a biomarker to predict good immune response in NRAS wildtype melanoma and guide immunotherapeutic responsiveness.

## Introduction

In melanoma, NRAS is one of the most significant driver genes and comprises 15%–20% of all melanomas (1). Correspondingly, NRAS wildtype patients, who account for 80%–85% of melanomas, have a better prognosis (2). Immune checkpoint inhibitors (ICIs) have shown remarkable promise as anticancer treatment (3). For patients with metastatic melanoma, monoclonal antibodies, which block CTLA-4 (Ipilimumab), PD-1 (Pembrolizumab, Nivolumab, and Toripalimab), or PD-L1 (Atezolizumab), have been approved as standard therapies (3–5).

Research has shown that TIL infiltration levels in NRAS wildtype melanoma are higher than in NRAS mutant melanoma (6). However, in clinical studies, the influence of NRAS gene mutation status (mutation or wildtype) on melanoma immunotherapy has not been decisively proven. Johnson et al. (1) revealed that the benefit of ICI treatment in advanced melanoma with NRAS mutation (NRAS-Mut) exceeded that in wildtype patients, especially from anti-PD-1/PD-L1 treatment (ORR 64% vs. 30%). However, the results from the POLARIS-01 study showed that NRAS mutation may be a potential resistance mechanism of immunotherapy in advanced melanoma patients (7). The previous pooled analysis of four clinical trials also found that patients with NRAS wildtype melanoma had significantly higher response rates and better prognoses than patients with NRAS-mutant melanoma following anti-PD-1 monotherapy (8). Based on the findings of these studies, it can be speculated that NRAS may be an important factor in melanoma that affects the efficacy of immunotherapy and that studies on biomarkers should use NRAS status as a classification factor for the melanoma population.

Biomarkers for ICI responses, such as programmed death-ligand 1 (PD-L1) expression and tumor mutation burden (TMB), have been extensively investigated in many types of cancer (9, 10). However, using PD-L1 expression levels and TMB as biomarkers to assist in immunotherapy selection for melanoma is controversial, as clinical benefits have also been observed in PD-L1-negative and TMB-low patients (11, 12). NRAS wildtype patients represent approximately 80%–85% of the entire melanoma population, and no research has been reported on the biomarker of ICI therapy for this population. Accordingly, the exploration of novel precise biomarkers and mechanisms in NRAS wildtype patients must maximize the clinical benefit.

The NOTCH pathway, is an extremely conserved signaling pathway, which is regulated by the short-range cell–cell interaction between NOTCH receptor (NOTCH1–4) and “canonical” ligand (Jagged1, Jagged2, DLL1, DLL3, or DLL4) (13); or noncanonically by activation of other pathways such as WNT, TGFβ, and NF-kB (14). The NOTCH family has been explicitly demonstrated to play a role in cellular proliferation, differentiation, apoptosis, and tumor invasion in various human cancers (15, 16). Some recent studies have demonstrated that the Notch signaling pathway is associated with tumor immunogenicity and anti-tumor immune efficacy. Notch signaling is crucial for various stages of T-cell development and differentiation (17, 18). A multiple-dimensional clinical data analysis has identified NOTCH1, 2, and 3 mutations as effective predictors of immunotherapy in NSCLC; however, NOTCH4 was excluded. In a study of small cell lung cancer, mutation of the NOTCH signal pathway was also associated with the benefit of ICI treatment (19). In a pan-cancer (lung cancer, cervical cancer, prostate cancer, pancreatic cancer, colon cancer, kidney cancer, large cell and Hodgkin’s lymphoma, etc.) biomarker research published recently, NOTCH4 may serve as a potential predictive biomarker for ICI treatment (20). Furthermore, several basic studies have confirmed that the NOTCH family genes play a crucial role in melanoma (15, 21). Nevertheless, the association between NOTCH family gene mutations and clinical benefit in NRAS wildtype melanoma immunotherapy remains unknown.

In this study, we implemented a comprehensive analysis of the immunotherapy predictive functions of NOTCH family genes (NOTCH1–4). We disclosed that mutated NOTCH4 in NRAS wildtype patients was a predictive biomarker for better outcomes of immunotherapy. The potential mechanism was subsequently explored through RNA expression in an immunotherapy treated population.

## Material and methods

### Clinical cohorts (discovery and validation cohort)

To evaluate the predictive value of all NOTCH family genes in ICIs-treated melanoma, we methodically collected whole-exome sequencing (WES) data and corresponding clinical information from the Allen (22) cohort. The processed mutation data were obtained from the cBioPortal website (https://www.cbioportal.org) (23, 24). All nonsynonymous somatic mutations, including nonsense, missense, nonstop, frameshift deletion and insertion, in-frame deletion and insertion, and splice site mutations, were considered for inclusion in our study (25). NOTCH4-mutant (NOTCH4-Mut) and NOTCH4-wildtype (NOTCH4-Wt) tumors were defined as having and without nonsynonymous somatic mutations of NOTCH4, respectively.

**Figure 1:**
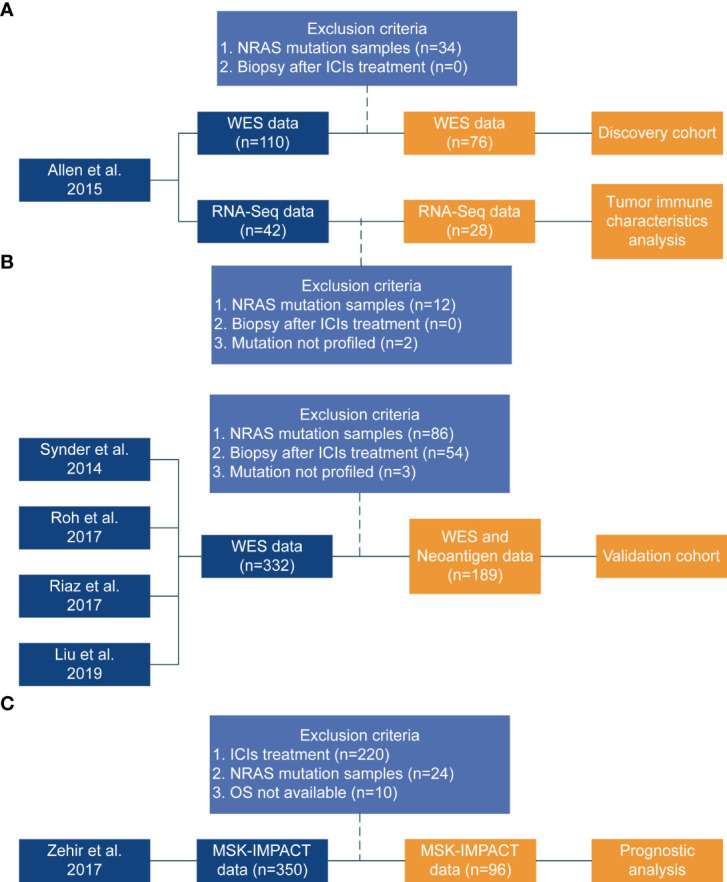
Flowchart of the study design. **(A)** Discovery cohort from published study [Allen et al. (22)]. **(B)** Coalition of the validation cohort from four published studies (Synder et al. (27), Roh et al. (28), Riaz et al. (26), Liu et al. (11)). The Synder, Roh, and Riaz study did not include PFS data. **(C)** Zehir study (29) data used to perform prognostic analysis.

We only used the data from pre-treatment biopsy samples because the mutation status of patients would change following ICI treatment (26). Additionally, samples that just had RNA expression data but no DNA-based mutation data (mutation not profiled) were excluded. Finally, the discovery cohort included 76 NRAS-wildtype melanoma. Twenty-eight NRAS wildtype RNA samples in this cohort can be used for analysis. Furthermore, we validated the predictive potency of NOTCH4-Mut in a combination of four independently public cohorts of 189 NRAS-wildtype melanoma that was composed of 25 patients from Synder et al. (27), 14 patients from Roh et al. (28), 53 patients from Riaz et al. (26), and 97 patients from Liu et al. (11). Processing and analyzing of the validation cohort are shown in **Figure 1**.

### Evaluation of prognostic value

The survival data of 96 NRAS wildtype patients without ICI treatment were retrieved from the Zehir cohort (29) was used to explore the prognostic influence of NOTCH4 (**Figure 1**).

### Clinical outcomes

The primary clinical outcomes were progression-free survival (PFS), overall survival (OS), and objective response rate (ORR). The tumor response for patients, including complete response (CR), partial response (PR), or stable disease (SD), was defined by RECIST or RECIST v1.1. PFS was defined as the date that the patient began ICI treatment to the date of progression or death of any cause. Patients without progression were censored at the date of their last scan. The OS was defined as from the date of ICI treatment in the immunotherapy cohort, or from the date of the first non-ICI (target-therapies/chemotherapies) treatment in the non-ICI treated cohort, respectively.

### The Cancer Genome Atlas (TCGA) cohort

Somatic mutations of 10,953 patients across 33 tumor types, including SKCM (n = 442), were retrieved from the UCSC Xena data portal (https://xenabrowser.net) (30), which were used for the analysis of NOTCH4-Mut frequency and mutation distribution. Nonsynonymous mutations in the coding region of the NOTCH4 gene were defined as NOTCH4 mutations. The MutationMapper module (https://www.cbioportal.org/mutation_mapper) from the cBioPortal was used to investigate the distribution of mutations in the protein domain.

### TMB and TNB analysis

TMB was defined as the total number of mutations per megabase (Mb) of genome with non-synonymous somatic, coding, base substitution, and indel variants and was calculated using maftools (31). For whole-exome sequencing data, 38 Mb was used as the estimated exome size in the discovery cohort and the validation cohort. Median TMB (<median, ≥median) was adopted as the cutoff value for high/low TMB in this study. The tumor neoantigen burden (TNB) of validation cohort samples was obtained from the cBioPortal web (https://www.cbioportal.org) in Roh et al. (28) and Riaz et al. (26) studies.

### Correlation analysis with the DDR pathway

The DNA damage response (DDR) pathway genes (**Supplementary Table S1**) were acquired from the Broad Institute Molecular Signatures Database (MSigDB) (32) to compare the differences in the mean mutation number of the DDR pathway between NOTCH4-Mut and NOTCH4-Wt in NRAS-wildtype melanoma.

### Correlation analysis for tumor immunogenicity and immune features

The CIBERSORT web portal (https://cibersort.stanford.edu/) was used to assess 22 types of infiltrating immune cells in the discovery cohort based on normalized gene expression data. The CIBERSORT immune infiltration proportions and immune-associated gene list were obtained from the pan-cancer immune landscape project implemented by Thorsson et al. (21). The expression levels of these immune-associated genes are quantified as log2 (FPKM + 1).

### Gene set enrichment analysis (GSEA)

GSEA was conducted using the Java GSEA 4.1.0 Desktop Application (http://www.gsea-msigdb.org/gsea/index.jsp) to identify whether immune-related gene signatures were associated with NOTCH mutation status (33). The R package DESeq2 was used to produce properly normalized RNA-seq Counts data, which are compatible with GSEA (34). The gene sets examined in GSEA of immunologic signature gene sets were obtained from the Molecular Signatures Database (MSigDB database v7.4) (http://www.gsea-msigdb.org/gsea/downloads.jsp). This analysis involved 1,000 random permutations for gene sets and a weighted enrichment statistic. The normalized enrichment score (NES) is the primary statistic for checking the GSEA results. The false discovery rate (FDR) was used to control the proportion of false-positives. FDR estimates the probability that a gene set with a prescribed NES represents a false-positive finding. A significantly enriched gene set was expected at FDR <0.05. FDR <0.05 was the significantly enriched gene set.

### Statistical analysis

Continuous variables were compared by the Mann–Whitney U test and categorical variables were compared using the chi-square test or Fisher’s exact test. The Kaplan–Meier curve (K-M curve) analysis of PFS and OS was compared by the log-rank test. The Cox proportional hazards regression was applied for univariable and multivariate analysis, and available confounding factors including sex, age, drug category, tumor stage, and TMB level were adjusted. Variables with *P <*0.05 in the univariable regression and those which have been reported to be linked with the efficacy of immunotherapy for melanoma were also included in the multivariable Cox regression. All reported p-values were two-tailed, and *p <*0.05 was considered statistically significant. Statistical analyses were performed using R v. 4.0.3 (https://www.r-project.org).

## Results

### NOTCH4-Mut predicts favorable clinical outcomes to immunotherapy in the discovery cohort

**Figure 2:**
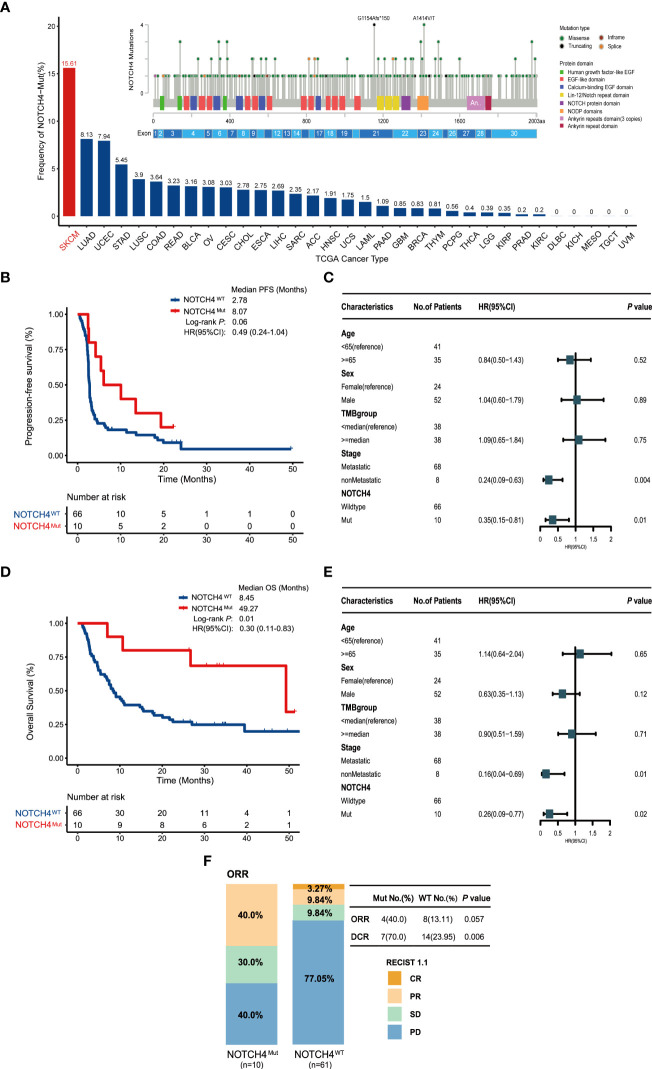
NOTCH4 mutation status and the association between NOTCH4 mutations and clinical outcomes for immunotherapy in the discovery cohort (N = 76). **(A)** The frequency of NOTCH4 mutations across 33 types of cancer in the TCGA. The numbers above the barplot indicate the alteration frequency. **(B)** Kaplan–Meier survival analysis comparing PFS between NOTCH4-Mut and NOTCH4-Wt patients in the discovery cohort. **(C)** Multivariate Cox regression analysis of NOTCH4 mutations with age, gender, stage (metastasis, means distant metastases, including IV stage with M1a, M1b, and M1c vs non-metastasis, means without distant metastases including stage IIIC and IV stage with M0) and TMB (<median vs ≥median= status were taken into account. Square data markers indicate estimated hazard ratios. Error bars represent 95% CIs. **(D)** Kaplan–Meier survival analysis comparing OS between NOTCH4-Mut and NOTCH4-Wt patients in the discovery cohort. **(E)** Multivariate Cox regression analysis of NOTCH4 mutations with age, gender, stage, and TMB status was taken into account. **(F)** The ratio of patients with complete response (CR), partial response (PR), stable disease (SD), and progression disease (PD) treated with ICIs in NOTCH4-Mut and NOTCH4-Wt groups. *P <*0.05 by Fisher’s exact test. There are 71 patients available to evaluate clinical benefit.

Detailed clinical information of the discovery cohort is summarized in **Supplementary Table S2**. The NOTCH family, including NOTCH1, NOTCH2, NOTCH3, and NOTCH4, was investigated. In the discovery cohort, 110 melanoma patients included 76 NRAS wildtype patients. Mutation frequencies of NOTCH1–4 in this subset are 4%, 11%, 5%, and 13%, respectively. The survival analysis of these four genes found that only the NOTCH4 mutation had better PFS and OS. The survival results of NOTCH1–3 are shown in **Supplementary Figure S1**. Additionally, the overall mutation frequency of NOTCH4 was 2.96% (324/10,953) in the TCGA pan-cancer cohort, with melanoma at 15.61% (69/442) ranking first (**Figure 2A**). The most frequent somatic mutation sites of NOTCH4 were G1154Afs*150 and A1414V/T, and usually all somatic variants were evenly distributed without any annotated functional cancer hotspot mutations from the Cancer Hotspots (35) (https://www.cancerhotspots.org) (**Figure 2A**).

Longer PFS was detected in NOTCH4-Mut melanoma (median PFS of 8.07 months vs. 2.78 months, HR = 0.49, 95% CI: 0.24–1.04; log-rank test *P* = 0.06, **Figure 2B**). After taking into account age, gender, stage, and TMB status, the result of multivariable Cox proportional hazards regression showed significantly better PFS (HR = 0.35, 95% CI: 0.15–0.81; *P* = 0.01, **Figure 2C**). As for OS analysis, NOTCH4-Mut patients achieved superior OS (median OS of 49.27 months vs. 8.45 months, HR = 0.30, 95% CI: 0.11–0.83; log-rank test *P* = 0.01, **Figure 2D**). After adjusting for the same confounding factors, a significant OS benefit still existed (HR = 0.26, 95% CI: 0.09–0.77; *P* = 0.02, **Figure 2E**). The proportion of ORR (CR + PR) in NOTCH4-Mut patients was three times that in the NOTCH4 wildtype group (40.0% vs. 13.11%, *P* = 0.057, **Figure 2F**). NOTCH4-Mut patients has tripled in DCR (CR + PR + SD) proportion (70.0% vs. 23.95%, *P* = 0.006, **Figure 2F**). These results indicated NOTCH4 might be a potential ICIs treatment biomarker.

### NOTCH4-Mut predicted clinical benefit in validation cohort

**Figure 3:**
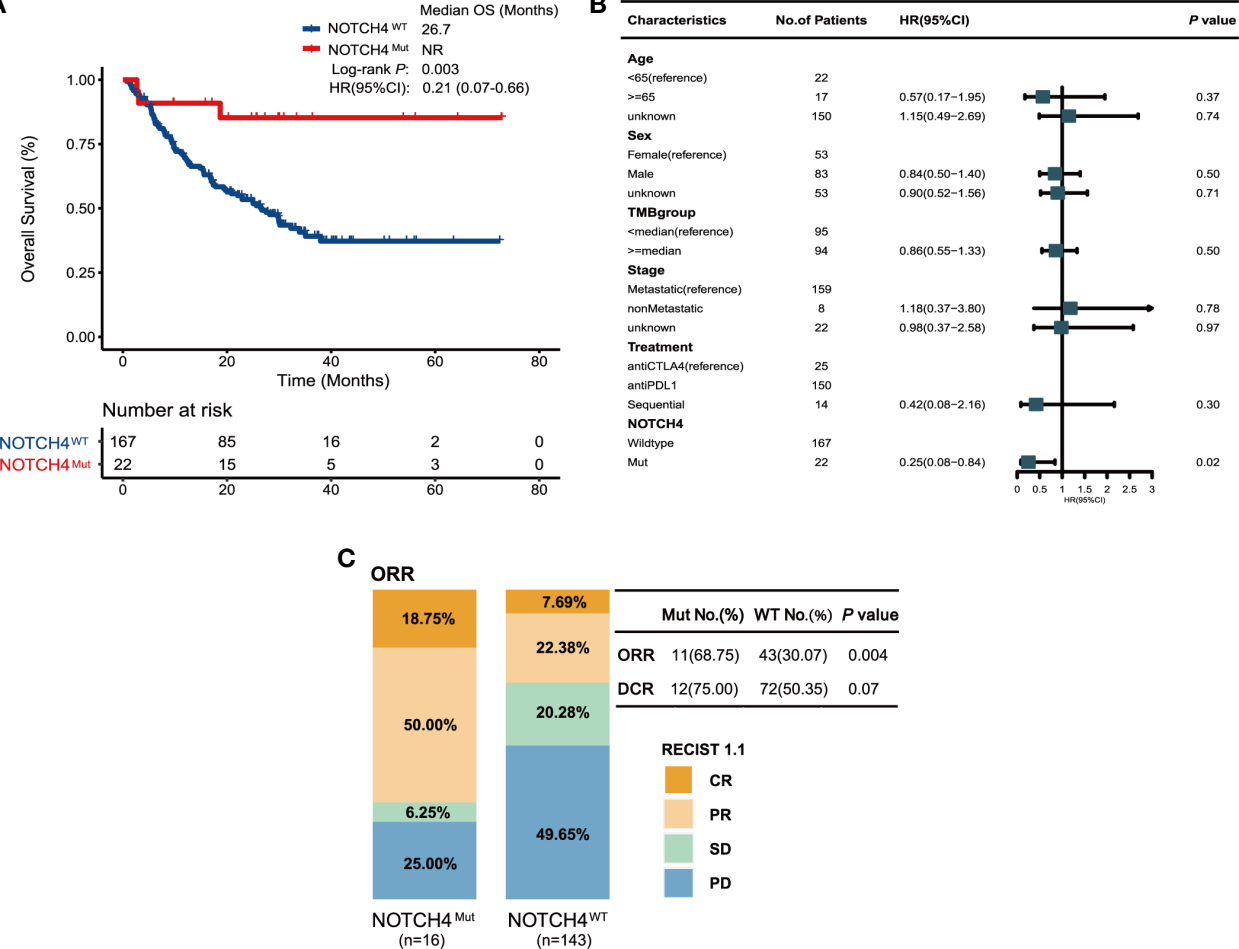
Validation of the predictive function of NOTCH4 mutations. **(A)** Kaplan–Meier survival analysis comparing OS between NOTCH4-Mut and NOTCH4-Wt patients in the validation cohort. NR, not reached. **(B)** Multivariate Cox regression analysis of NOTCH4 mutations with age, gender, stage (metastasis, means distant metastases, including IV stage with M1a, M1b, and M1c vs non-metastasis, means without distant metastases including IIIC stage and IV stage with M0) and TMB status were considered. Square data markers indicate estimated hazard ratios. Error bars represent 95% CIs. **(C)** The ratio of patients with complete response (CR), partial response (PR), stable disease (SD), and progression disease (PD) treated with ICIs in NOTCH4-Mut and NOTCH4-Wt group. *P <*0.05 by Fisher’s exact test. There are 159 patients available to evaluate clinical benefit.

To further investigate the survival benefit in ICIs-treated NRAS wildtype melanoma with NOTCH4 mutation, the survival analysis was performed in a combination of four published clinical ICI treatment cohort (11, 26–28). A total of 189 NRAS wildtype melanoma were enrolled in the validation cohort, and the NOTCH4 mutation frequency in the validation cohort was 12% (22/189). Detailed clinical information of the validation cohort is summarized in **Supplementary Table S2**. The survival analysis found that NOTCH4-Mut melanomas had better OS than NOTCH-Wt patients (median OS was not reached, NR months vs. 26.7 months, HR = 0.21, 95% CI: 0.07–0.66; Log rank test *P* = 0.003, **Figure 3A**). In the multivariable Cox proportional hazards regression model adjusted as the same as the discovery cohort by age, gender, stage, and TMB status, numerical OS superiority still existed (HR = 0.25, 95% CI: 0.08–0.84; *P* = 0.02, **Figure 3B**). The specific analysis of ORR and DCR between NOTCH4-Mut and NOTCH4-Wt was also explored. Compared with wildtype patients, NOTCH4-Mut patients has doubled in ORR proportion (68.75% vs. 30.07%, *P* = 0.004, **Figure 3C**). The proportion of DCR in NOTCH4-Mut patients was higher than in NOTCH4-Wt patients (75.0% vs. 50.35%, *P* = 0.07, **Figure 3C**).

### NOTCH4-Mut patients had a higher TMB and TNB

**Figure 4:**
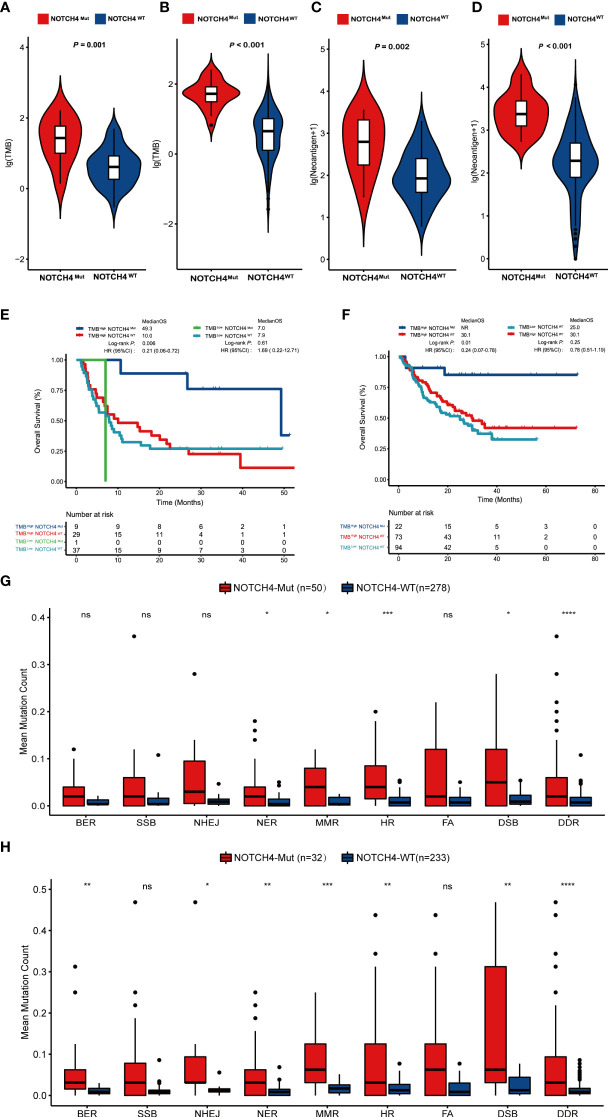
NOTCH4-Mut patients have an association with TMB and TNB. **(A,B)** Comparing TMB with NOTCH4-Mut and NOTCH4-Wt patients in the discovery cohort **(A)** and validation cohort **(B)**. **(C, D)** Comparing TNB with NOTCH4-Mut and NOTCH4-Wt patients in the discovery cohort **(C)** and validation cohort **(D)**. **(E, F)** The Kaplan–Meier curve comparing OS among NOTCH4^Mut^TMB^High^, NOTCH4^Wt^TMB^High^, NOTCH4^Mut^TMB^Low^, and NOTCH4^Wt^TMB^Low^ group in discovery cohort **(E)** and validation cohort **(F)**. **(G,H)** Comparison of DNA damage-related gene set variants between NOTCH4-Mut and NOTCH4-Wt groups from the TCGA NRAS-wildtype melanoma samples **(G)** and the combination NRAS-wildtype melanoma of the discovery and validation cohorts **(H)**. BER, Base Excision Repair; SSB, Single-Strand Breaks; NHEJ, Non-Homologous End Joining; NER, Nucleotide Excision Repair; MMR, Mis-Match Repair; HR, Homologous Recombination; FA, Fanconi Anemia; DSB, Double-Strand Breaks, DDR, DNA damage response. Notes: *P < 0.05; **P < 0.01; ***P < 0.001; ****P < 0.0001; ns, P < 0.05.

We further examined whether the NOTCH4 mutations were preferentially associated with TMB and TNB, which are considered to be the common markers of pan-cancer immunotherapy. The result showed the NOTCH4-Mut group had significantly higher TMB and TNB both in discovery and validation cohorts (**Figures 4A–D**). Therefore, according to NOTCH4 status and TMB levels, we divided patients into four groups: NOTCH4^Mut^TMB^High^, NOTCH4^Wt^TMB^High^, NOTCH4^Mut^TMB^Low^, and NOTCH4^Mut^TMB^Low^. As expected, NOTCH4^Mut^TMB^High^ patients had the longest OS among all groups, both in the discovery (**Figure 4E**) and in the validation cohorts (**Figure 4F**). In the discovery cohort, notably, despite having a high TMB, patients with the NOTCH4 mutation lived substantially longer following immunotherapy than those with the wildtype mutation (49.3 vs. 10.0 months, HR = 0.21, 95% CI: 0.06–0.72, *P* = 0.006). Similar findings were obtained from the validation cohort (median OS: NR vs. 30.1 months, HR = 0.24, 95% CI: 0.07–0.78, *P* = 0.01). Interestingly, all NOTCH-Mut patients were in the NOTCH4^Mut^TMB^High^ group in the validation cohort. While high TMB is associated with multiple factors, including DDR gene alterations. Therefore, we used the DDR gene sets (**Supplementary Table S1**) from MSigDB to explore the differences in DDR pathway mutations between NOTCH4-Mut and NOTCH4-Wt tumors in NRAS-wildtype melanoma. In the TCGA cohort, NOTCH4-Mut tumors had a meaningfully increased number of DDR mutations (**Figure 4G**). In the immunotherapy cohort, DDR pathway gene mutations in NOTCH4-Mut samples were also greater (**Figure 4H**).

### NOTCH4 is not a prognosis factor

To evaluate the prognostic value of NOTCH4, survival analysis was further conducted according to NOTCH4 status in a non-ICI treated cohort. The percentage of NOTCH4 mutations is 9.4% (9/96) in NRAS wildtype melanoma. Although NOTCH4-Mut patients seemed to have a long survival trend, no significant difference was found in OS between NOTCH4-Mut and NOTCH4-Wt subsets in melanoma patients (both median OS were NR, *P* = 0.27, **Supplementary Figure S2**). Therefore, NOTCH4 mutation is not a prognosis biomarker of ICI benefit.

### Potential mechanisms associated with NOTCH4 mutation in predicting immunotherapy efficacy in NRAS wildtype tumors

**Figure 5:**
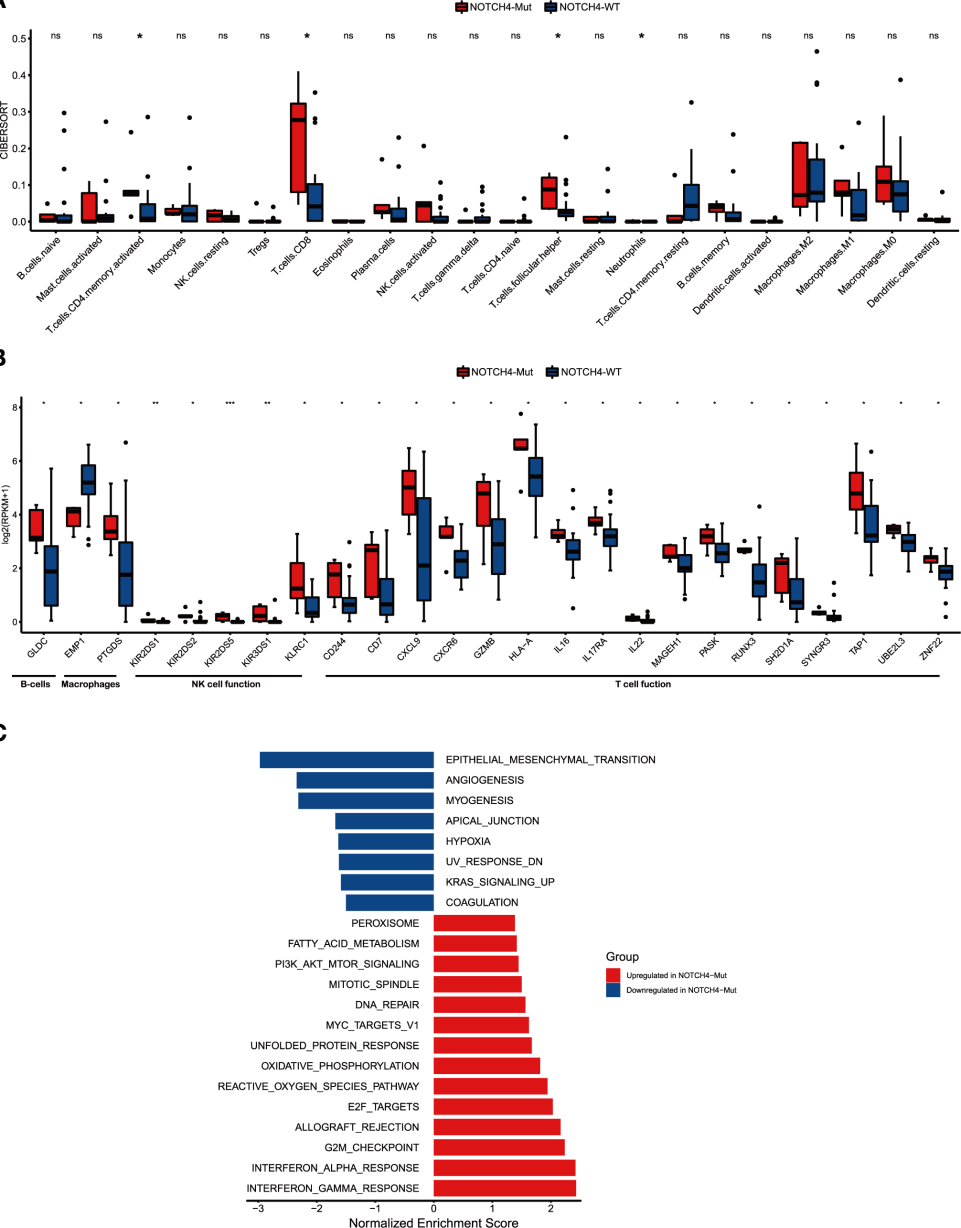
NOTCH4-Mut was associated with enhanced anti-tumor immunity. **(A)** Boxplot depicting the infiltration of 22 immune cells in NOTCH4-Mut and NOTCH4-Wt tumors. Gene expression profiles were uploaded to the CIBERSORT web portal, and the algorithm was configured with 1,000 permutations. **(B)** Box plot of gene expression of immune-related genes in comparison of NOTCH4-Mut and NOTCH4-Wt patients. **(C)** Differences in pathway activities scored by GSEA Hallmark collection between NOTCH4-Mut and NOTCH4-Wt tumors. Enrichment results with significant associations between NOTCH4-Mut and NOTCH4-Wt tumors are shown (*P <*0.05, FDR <0.05). The blue bar indicates that the normalized enrichment score (NES) of the pathway is less than 0, while the red bar indicates that the NES of the pathway is more than 0. Notes: *P < 0.05; **P < 0.01; ***P < 0.001; ns, P > 0.05.

To further explore the fundamental mechanism of the predictive values of NOTCH4 mutations to immunotherapy efficacy in NRAS wildtype melanoma, the RNA data of 28 patients from the Allen study (22) was used. First, the CIBERSORT was used to explore the infiltration of immune cells, and the results are shown in **Figure 5A**. Enhanced anti-tumor immunity was observed in NOTCH4-Mut tumors. Activated CD4 memory T cells, CD8 T cells, follicular helper T cells (Tfh), and neutrophils were more abundant in NOTCH4-Mut tumors.

Studies have shown that in the TME, the Notch pathway is involved in cell lineage specification in developing lymphocytes and in the regulation of B lymphocyte subsets of the marginal zone, as well as differentiation and functions of dendritic cells and innate lymphoid cells, and helper and regulatory T cells (13). Given the link between NOTCH4 mutation and the result of infiltration of immune cells and the literature, we further investigated whether NOTCH4 mutation was associated with immune-related function gene signatures (**Figure 5B**). Of the 25 immune-related genes, 24 genes (96%) demonstrated higher expression levels in NOTCH4-Mut tumors compared with wildtype ones, but one gene (4%), EMP1, showed decreased expression, implying a potential decrease in cancer invasiveness and metastasis. Additionally, the results of enrichment analysis indicated that several pathways varied significantly between NOTCH4-Mut and NOTCH4-Wt tumors, including interferon γ response, DNA repair, epithelial–mesenchymal transition (EMT), angiogenesis, and other biological functions (**Figure 5C**). These signaling pathways are closely related to immunotherapy.

## Discussion

NRAS mutant melanoma has been the focus of researchers in both targeted therapy and immunotherapy. Among melanoma patients, NRAS wildtype patients account for about 80% of all cases. Exploration of immunotherapy biomarkers for these patients could bring benefit to more melanoma patients. In our research, we comprehensively converged and consolidated both genomic and clinical data to evaluate the relationship between NOTCH4 mutation and the outcome of an ICI-treated NRAS wildtype melanoma cohort. Then we seriously validated our findings in a combination of four independent clinical cohorts. Actually, we also performed additional analysis of NOTCH4 predictive value within NRAS mutant melanoma both in the discovery and validation cohorts. In NRAS mutant patients of the discovery cohort, NOTCH4-Mut melanoma had longer OS (mOS: 34.93 months vs. 6.43 months, HR = 0.37, 95% CI: 0.09–1.61, *P* = 0.17) than wildtype melanoma, but there was no statistical difference. In NRAS mutant patients of the validation cohort, NOTCH4 wildtype melanoma had longer OS (mOS: 31.3 months vs. 22.6 months, HR = 1.23, 95% CI: 0.48–3.18, *P* = 0.68) than NOTCH4-Mut patients (**Supplementary Figure S3**). Given the different clinical studies (1, 7) and our analysis results, the role of NRAS in immunotherapy is controversial and complex. More basic research is needed to elucidate this mechanism. Therefore, NOTCH4 is a superior predictive biomarker in NRAS wildtype melanoma. For instance, patients with NOTCH4 mutations exhibited higher TMB, TNB, and numbers of DDR pathway mutations, compared with patients with wildtype tumors, indicating increased tumor immunogenicity. Analysis of the infiltration of immune cells and pathway enrichment analysis also confirmed NOTCH4 mutations increased tumor immunity. To our best knowledge, this study is the first to propose that NOTCH4 mutation might be a predictor of favorable immunotherapy in NRAS wildtype melanoma.

The Notch signaling pathway is an important and complex pathway that is recognized as a major actor both in cancer cells and in each component of the tumor microenvironment (TME) (17, 36). At present, there are some studies on the prediction of NSCLC immunotherapy by the NOTCH signaling pathway, but we found that its prediction effect of immunotherapy is different. Zhang et al. (37) presented harmful NOTCH1/2/3 mutations rather than NOTCH4 could be a predictor for efficacious immunotherapy in NSCLC. Li et al. (38) found that high-mutated NOTCH signaling (the NOTCH signaling gene set was derived from the MSigDB) could serve as an independent predictor of NSCLC patients receiving ICIs. Interestingly, when we were writing our finding that the association between NOTCH4 mutation and ICI response was established, we found a study in which NOTCH4 predicted immune efficacy in pan-cancer, including NSCLC and melanoma (20). In that study, the NOTCH4 mutation had a predictive value of melanoma in the discovery cohort. However, there were no OS differences between NOTCH4-Mut and NOTCH4-Wt melanoma patients in the validation cohort.

We conjectured that the reason that the study failed to verify might not distinguishing the population of the driver mutation subpopulation. Our study further demonstrated the predictive role of NOTCH4 in melanoma immunotherapy and identified the benefit group as wildtype NRAS melanoma patients. Additionally, we elucidated for the first time the relevance of NOTCH4 mutations to the DDR pathway in melanoma. The DDR pathway is important for maintaining genomic stability and integrity. Mutations in the DDR pathway would increase genomic instability and TMB. Many studies have shown that mutations in the DDR pathway could serve as a predictive biomarker for immunotherapy (39–41). Consequently, more DDR pathway mutations in NOTCH4-Mut melanoma may be a reason why immunotherapies are more effective in these patients. Based on the pan-cancer study and our specific NRAS wildtype melanoma research, we believe that NOTCH4 can be a promising ICI biomarker and is worthy of clinical verification.

However, our retrospective analysis also has several limitations. First, although NOTCH4 mutation frequency is above 10% in both the discovery and validation cohorts, the sample size of the NOTCH4-Mut samples in the discovery cohort (n = 10) or in the validation cohort (n = 22) is relatively small. More prospective studies are urgent to determine the efficiency of NOTCH4 in NRAS widetype melanoma. Second, although NOTCH4-Mut melanoma is characterized by infiltration of some T cell immune cells and some immune-related pathways, including interferon γ response, DNA repair, epithelial–mesenchymal transition, angiogenesis, and so on, the mechanistic underpinnings of NOTCH4 mutation relevant to ICI response remain elusive and merit further experimental work. Finally, all the cohort data we used are showed most cutaneous melanoma. The predictive performance of NOTCH4 in acral lentiginous melanoma and mucosal melanoma needs to be further explored.

## Conclusion

In summary, our study indicates the favorable relationship between NOTCH4 mutation and better clinical outcomes in ICI treatment NRAS wildtype melanoma patients. Therefore, NOTCH4 could serve as a predictive biomarker for NRAS wildtype patients. Furthermore, validation of NOTCH4 predictive value in prospective trials and more exploration of its molecular mechanism are needed in the future.

## Data availability statement

The datasets presented in this study can be found in online repositories. The names of the repository/repositories and accession number(s) can be found in the article/**Supplementary Material**.

## Author contributions

(I) Conception and design: HL. (II) Administrative support: TS and CQ. (III) Provision of study materials or patients: HL and QZ. (IV) Collection and assembly of data: QZ, QD, and YT. (V) Data analysis and interpretation: QZ, QD, and YT. (VI) Manuscript writing: All authors. (VII) Final approval of manuscript: All authors

## Conflict of interest

Authors QZ, QD, YT, TS, and CQ were employed by Jiangsu Simcere Diagnostics Co., Ltd., the Nanjing Simcere Medical Laboratory Science Co., Ltd., and The State Key Lab of Translational Medicine and Innovative Drug Development, Jiangsu Simcere Diagnostics Co., Ltd.

The remaining author declares that the research was conducted in the absence of any commercial or financial relationships that could be construed as a potential conflict of interest.

## Publisher’s note

All claims expressed in this article are solely those of the authors and do not necessarily represent those of their affiliated organizations, or those of the publisher, the editors and the reviewers. Any product that may be evaluated in this article, or claim that may be made by its manufacturer, is not guaranteed or endorsed by the publisher.

## Supplementary material

Supplementary Figure 1 The survival results of NOTCH1, NOTCH2, NOTCH3 from discovery cohort. **(A)** The Kaplan-Meier survival analysis comparing PFS between NOTCH1-Mut and NOTCH1-Wt patients. **(B)** The Kaplan-Meier survival analysis comparing OS between NOTCH1-Mut and NOTCH1-Wt patients. **(C)** The Kaplan-Meier survival analysis comparing PFS between NOTCH2-Mut and NOTCH2-Wt patients. **(D)** The Kaplan-Meier survival analysis comparing OS between NOTCH2-Mut and NOTCH2-Wt patients. **(E)** The Kaplan-Meier survival analysis comparing PFS between NOTCH3-Mut and NOTCH3-Wt patients. **(F)** The Kaplan-Meier survival analysis comparing OS between NOTCH3-Mut and NOTCH3-Wt patients.

Supplementary Figure 2 The Kaplan-Meier curves of OS between NOTCH4-Mut and NOTCH4-Wt group in the non-ICIs cohort from Zehir cohort.

Supplementary Figure 3 The Kaplan-Meier curves of OS between NOTCH4-Mut and NOTCH4-Wt group in NRAS mutant melanoma of discovery cohort **(A)** and validation cohort **(B)**.

## References

[1] Johnson DB Lovly CM Flavin M Panageas KS Ayers GD Zhao Z. Impact of NRAS mutations for patients with advanced melanoma treated with immune therapies. Cancer Immunol Res (2015) 3(3):288–95. doi: 10.1158/2326-6066.CIR-14-0207 PMC4351797 25736262

[2] Jakob JA Bassett RL Jr. Ng CS Curry JL Joseph RW Alvarado GC. NRAS mutation status is an independent prognostic factor in metastatic melanoma. Cancer (2012) 118(16):4014–23. doi: 10.1002/cncr.26724 PMC3310961 22180178

[3] Bagchi S Yuan R Engleman EG. Immune checkpoint inhibitors for the treatment of cancer: Clinical impact and mechanisms of response and resistance. Annu Rev Pathol (2021) 16:223–49. doi: 10.1146/annurev-pathol-042020-042741 33197221

[4] Larkin J Chiarion-Sileni V Gonzalez R Grob JJ Rutkowski P Lao CD. Five-year survival with combined nivolumab and ipilimumab in advanced melanoma. N Engl J Med (2019) 381(16):1535–46. doi: 10.1056/NEJMoa1910836 31562797

[5] Hamid O Robert C Daud A Hodi FS Hwu WJ Kefford R. Five-year survival outcomes for patients with advanced melanoma treated with pembrolizumab in KEYNOTE-001. Ann Oncol (2019) 30(4):582–8. doi: 10.1093/annonc/mdz011 PMC6503622 30715153

[6] Thomas NE Edmiston SN Alexander A Groben PA Parrish E Kricker A. Association between NRAS and BRAF mutational status and melanoma-specific survival among patients with higher-risk primary melanoma. JAMA Oncol (2015) 1(3):359–68. doi: 10.1001/jamaoncol.2015.0493 PMC4486299 26146664

[7] Tang B Chi Z Chen Y Liu X Wu D Chen J. Safety, efficacy, and biomarker analysis of toripalimab in previously treated advanced melanoma: Results of the POLARIS-01 multicenter phase II trial. Clin Cancer Res (2020) 26(16):4250–9. doi: 10.1158/1078-0432.Ccr-19-3922 32321714

[8] Zhou L Wang X Chi Z Sheng X Kong Y Mao L. Association of NRAS mutation with clinical outcomes of anti-PD-1 monotherapy in advanced melanoma: A pooled analysis of four Asian clinical trials. Front Immunol (2021) 12:691032. doi: 10.3389/fimmu.2021.691032 34290710 PMC8289467

[9] Gibney GT Weiner LM Atkins MB. Predictive biomarkers for checkpoint inhibitor-based immunotherapy. Lancet Oncol (2016) 17(12):e542–51. doi: 10.1016/s1470-2045(16)30406-5 PMC5702534 27924752

[10] Chan TA Yarchoan M Jaffee E Swanton C Quezada SA Stenzinger A. Development of tumor mutation burden as an immunotherapy biomarker: Utility for the oncology clinic. Ann Oncol (2019) 30(1):44–56. doi: 10.1093/annonc/mdy495 30395155 PMC6336005

[11] Liu D Schilling B Liu D Sucker A Livingstone E Jerby-Arnon L. Integrative molecular and clinical modeling of clinical outcomes to PD1 blockade in patients with metastatic melanoma. Nat Med (2019) 25(12):1916–27. doi: 10.1038/s41591-019-0654-5 PMC6898788 31792460

[12] Gao Y Yang C He N Zhao G Wang J Yang Y. Integration of the tumor mutational burden and tumor heterogeneity identify an immunological subtype of melanoma with favorable survival. Front Oncol (2020) 10:571545. doi: 10.3389/fonc.2020.571545 33194669 PMC7661856

[13] Radtke F MacDonald HR Tacchini-Cottier F. Regulation of innate and adaptive immunity by notch. Nat Rev Immunol (2013) 13(6):427–37. doi: 10.1038/nri3445 23665520

[14] Ayaz F Osborne BA. Non-canonical notch signaling in cancer and immunity. Front Oncol (2014) 4:345. doi: 10.3389/fonc.2014.00345 25538890 PMC4255497

[15] Lin X Sun B Zhu D Zhao X Sun R Zhang Y. Notch4+ cancer stem-like cells promote the metastatic and invasive ability of melanoma. Cancer Sci (2016) 107(8):1079–91. doi: 10.1111/cas.12978 PMC4982579 27234159

[16] Ohnuki H Jiang K Wang D Salvucci O Kwak H Sanchez-Martin D. Tumor-infiltrating myeloid cells activate Dll4/Notch/TGF-beta signaling to drive malignant progression. Cancer Res (2014) 74(7):2038–49. doi: 10.1158/0008-5472.CAN-13-3118 PMC3985086 24520074

[17] Kelliher MA Roderick JE. NOTCH signaling in T-Cell-Mediated anti-tumor immunity and T-Cell-Based immunotherapies. Front Immunol (2018) 9:1718. doi: 10.3389/fimmu.2018.01718 30967879 PMC6109642

[18] Tchekneva EE Goruganthu MUL Uzhachenko RV Thomas PL Antonucci A Chekneva I. Determinant roles of dendritic cell-expressed notch delta-like and jagged ligands on anti-tumor T cell immunity. J Immunother Cancer (2019) 7(1):95. doi: 10.1186/s40425-019-0566-4 30940183 PMC6446314

[19] Roper N Velez MJ Chiappori A Kim YS Wei JS Sindiri S. Notch signaling and efficacy of PD-1/PD-L1 blockade in relapsed small cell lung cancer. Nat Commun (2021) 12(1):3880. doi: 10.1038/s41467-021-24164-y 34162872 PMC8222224

[20] Long J Wang D Yang X Wang A Lin Y Zheng M. Identification of NOTCH4 mutation as a response biomarker for immune checkpoint inhibitor therapy. BMC Med (2021) 19(1):154. doi: 10.1186/s12916-021-02031-3 34284787 PMC8293505

[21] Thorsson V Gibbs DL Brown SD Wolf D Bortone DS Ou Yang TH. The immune landscape of cancer. Immunity (2018) 48(4):812–30.e814. doi: 10.1016/j.immuni.2018.03.023 29628290 PMC5982584

[22] Van Allen EM Miao D Schilling B Shukla SA Blank C Zimmer L. Genomic correlates of response to CTLA-4 blockade in metastatic melanoma. Science (2015) 350(6257):207–11. doi: 10.1126/science.aad0095 PMC5054517 26359337

[23] Cerami E Gao J Dogrusoz U Gross BE Sumer SO Aksoy BA. The cbio cancer genomics portal: An open platform for exploring multidimensional cancer genomics data. Cancer Discov (2012) 2(5):401–4. doi: 10.1158/2159-8290.Cd-12-0095 PMC3956037 22588877

[24] Gao J Aksoy BA Dogrusoz U Dresdner G Gross B Sumer SO. Integrative analysis of complex cancer genomics and clinical profiles using the cbioportal. Sci Signal (2013) 6(269):pl1. doi: 10.1126/scisignal.2004088 23550210 PMC4160307

[25] Zhang L Han X Shi Y. Association of MUC16 mutation with response to immune checkpoint inhibitors in solid tumors. JAMA Netw Open (2020) 3(8):e2013201. doi: 10.1001/jamanetworkopen.2020.13201 32845327 PMC7450349

[26] Riaz N Havel JJ Makarov V Desrichard A Urba WJ Sims JS. Tumor and microenvironment evolution during immunotherapy with nivolumab. Cell (2017) 171(4):934–49.e916. doi: 10.1016/j.cell.2017.09.028 29033130 PMC5685550

[27] Snyder A Makarov V Merghoub T Yuan J Zaretsky JM Desrichard A. Genetic basis for clinical response to CTLA-4 blockade in melanoma. N Engl J Med (2014) 371(23):2189–99. doi: 10.1056/NEJMoa1406498 PMC4315319 25409260

[28] Roh W Chen PL Reuben A Spencer CN Prieto PA Miller JP. Integrated molecular analysis of tumor biopsies on sequential CTLA-4 and PD-1 blockade reveals markers of response and resistance. Sci Transl Med (2017) 9 (379):eaah3560. doi: 10.1126/scitranslmed.aah3560 28251903 PMC5819607

[29] Zehir A Benayed R Shah RH Syed A Middha S Kim HR. Mutational landscape of metastatic cancer revealed from prospective clinical sequencing of 10,000 patients. Nat Med (2017) 23(6):703–13. doi: 10.1038/nm.4333 PMC5461196 28481359

[30] Goldman MJ Craft B Hastie M Repečka K McDade F Kamath A. Visualizing and interpreting cancer genomics data *via* the xena platform. Nat Biotechnol (2020) 38(6):675–8. doi: 10.1038/s41587-020-0546-8 PMC7386072 32444850

[31] Mayakonda A Lin DC Assenov Y Plass C Koeffler HP. Maftools: Efficient and comprehensive analysis of somatic variants in cancer. Genome Res (2018) 28(11):1747–56. doi: 10.1101/gr.239244.118 PMC6211645 30341162

[32] Liberzon A Birger C Thorvaldsdóttir H Ghandi M Mesirov JP Tamayo P. The molecular signatures database (MSigDB) hallmark gene set collection. Cell Syst (2015) 1(6):417–25. doi: 10.1016/j.cels.2015.12.004 PMC4707969 26771021

[33] Subramanian A Tamayo P Mootha VK Mukherjee S Ebert BL Gillette MA. Gene set enrichment analysis: A knowledge-based approach for interpreting genome-wide expression profiles. Proc Natl Acad Sci USA (2005) 102(43):15545–50. doi: 10.1073/pnas.0506580102 PMC1239896 16199517

[34] Love MI Huber W Anders S. Moderated estimation of fold change and dispersion for RNA-seq data with Deseq2. Genome Biol (2014) 15(12):550. doi: 10.1186/s13059-014-0550-8 25516281 PMC4302049

[35] Chang MT Bhattarai TS Schram AM Bielski CM Donoghue MTA Jonsson P. Accelerating discovery of functional mutant alleles in cancer. Cancer Discov (2018) 8(2):174–83. doi: 10.1158/2159-8290.Cd-17-0321 PMC5809279 29247016

[36] Tsukumo SI Yasutomo K. Regulation of CD8(+) T cells and antitumor immunity by notch signaling. Front Immunol (2018) 9:101. doi: 10.3389/fimmu.2018.00101 29441071 PMC5797591

[37] Zhang K Hong X Song Z Xu Y Li C Wang G. Identification of deleterious NOTCH mutation as novel predictor to efficacious immunotherapy in NSCLC. Clin Cancer Res (2020) 26(14):3649–61. doi: 10.1158/1078-0432.CCR-19-3976 32241817

[38] Li X Wang Y Li X Feng G Hu S Bai Y. The impact of NOTCH pathway alteration on tumor microenvironment and clinical survival of immune checkpoint inhibitors in NSCLC. Front Immunol (2021) 12:638763. doi: 10.3389/fimmu.2021.638763 34305884 PMC8302260

[39] Wang Z Zhao J Wang G Zhang F Zhang Z Zhang F. Comutations in DNA damage response pathways serve as potential biomarkers for immune checkpoint blockade. Cancer Res (2018) 78(22):6486–96. doi: 10.1158/0008-5472.Can-18-1814 30171052

[40] Teo MY Seier K Ostrovnaya I Regazzi AM Kania BE Moran MM. Alterations in DNA damage response and repair genes as potential marker of clinical benefit from PD-1/PD-L1 blockade in advanced urothelial cancers. J Clin Oncol (2018) 36(17):1685–94. doi: 10.1200/jco.2017.75.7740 PMC6366295 29489427

[41] Jiang M Jia K Wang L Li W Chen B Liu Y. Alterations of DNA damage response pathway: Biomarker and therapeutic strategy for cancer immunotherapy. Acta Pharm Sin B (2021) 11(10):2983–94. doi: 10.1016/j.apsb.2021.01.003 PMC8546664 34729299

