# Supplementary figures and images for: NOTCH4 mutation as predictive biomarker for immunotherapy benefits in NRAS wildtype melanoma
Source: Front Immunol. 2022 Jul 29;13:894110. doi: 10.3389/fimmu.2022.894110 (PMC9372281; doi:10.3389/fimmu.2022.894110)

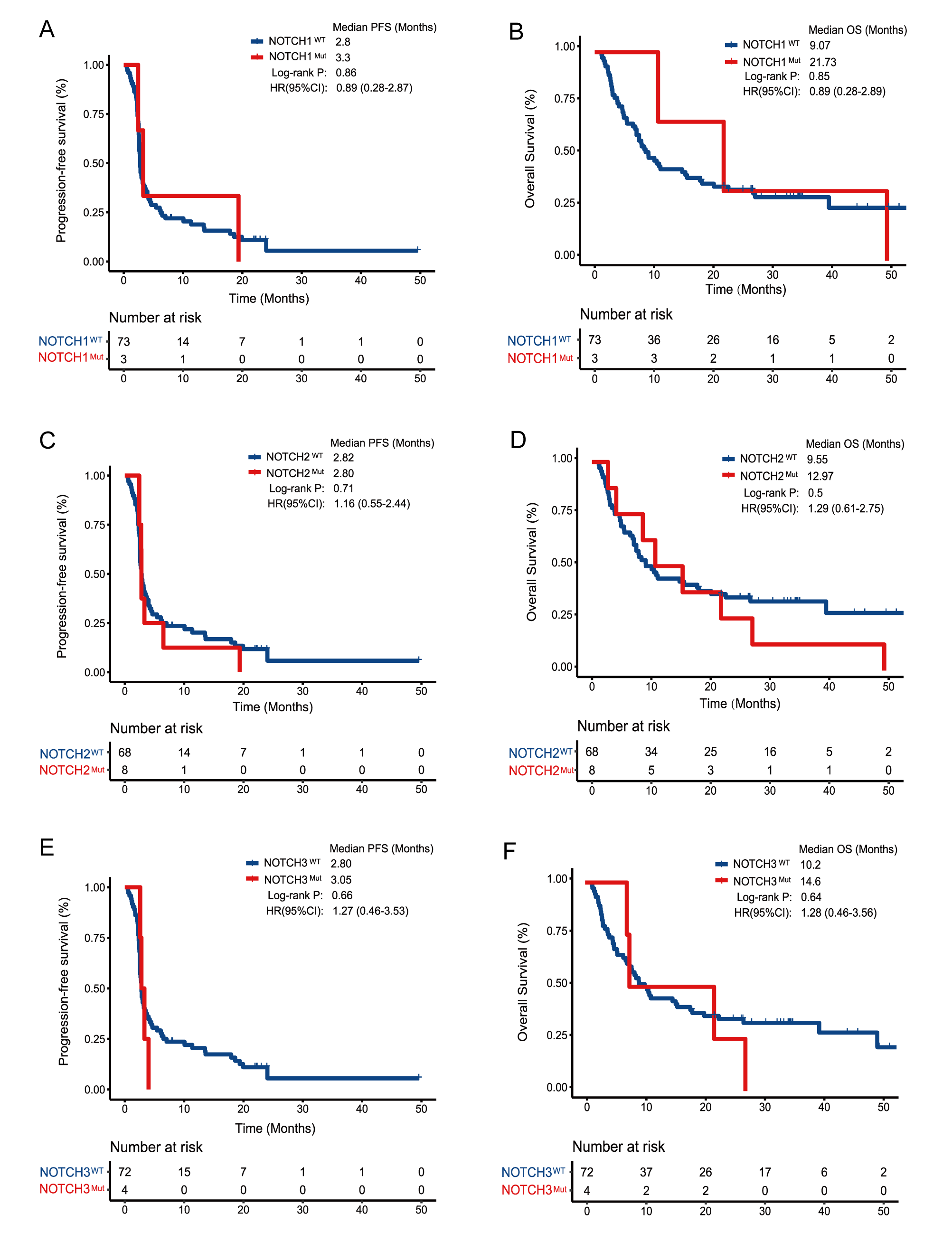

Supplement: Supplementary Figure 1 — The survival results of NOTCH1, NOTCH2, NOTCH3 from discovery cohort. (A) The Kaplan-Meier survival analysis comparing PFS between NOTCH1-Mut and NOTCH1-Wt patients. (B) The Kaplan-Meier survival analysis comparing OS between NOTCH1-Mut and NOTCH1-Wt patients. (C) The Kaplan-Meier survival analysis comparing PFS between NOTCH2-Mut and NOTCH2-Wt patients. (D) The Kaplan-Meier survival analysis comparing OS between NOTCH2-Mut and NOTCH2-Wt patients. (E) The Kaplan-Meier survival analysis comparing PFS between NOTCH3-Mut and NOTCH3-Wt patients. (F) The Kaplan-Meier survival analysis comparing OS between NOTCH3-Mut and NOTCH3-Wt patients. [file Image_1.tif]

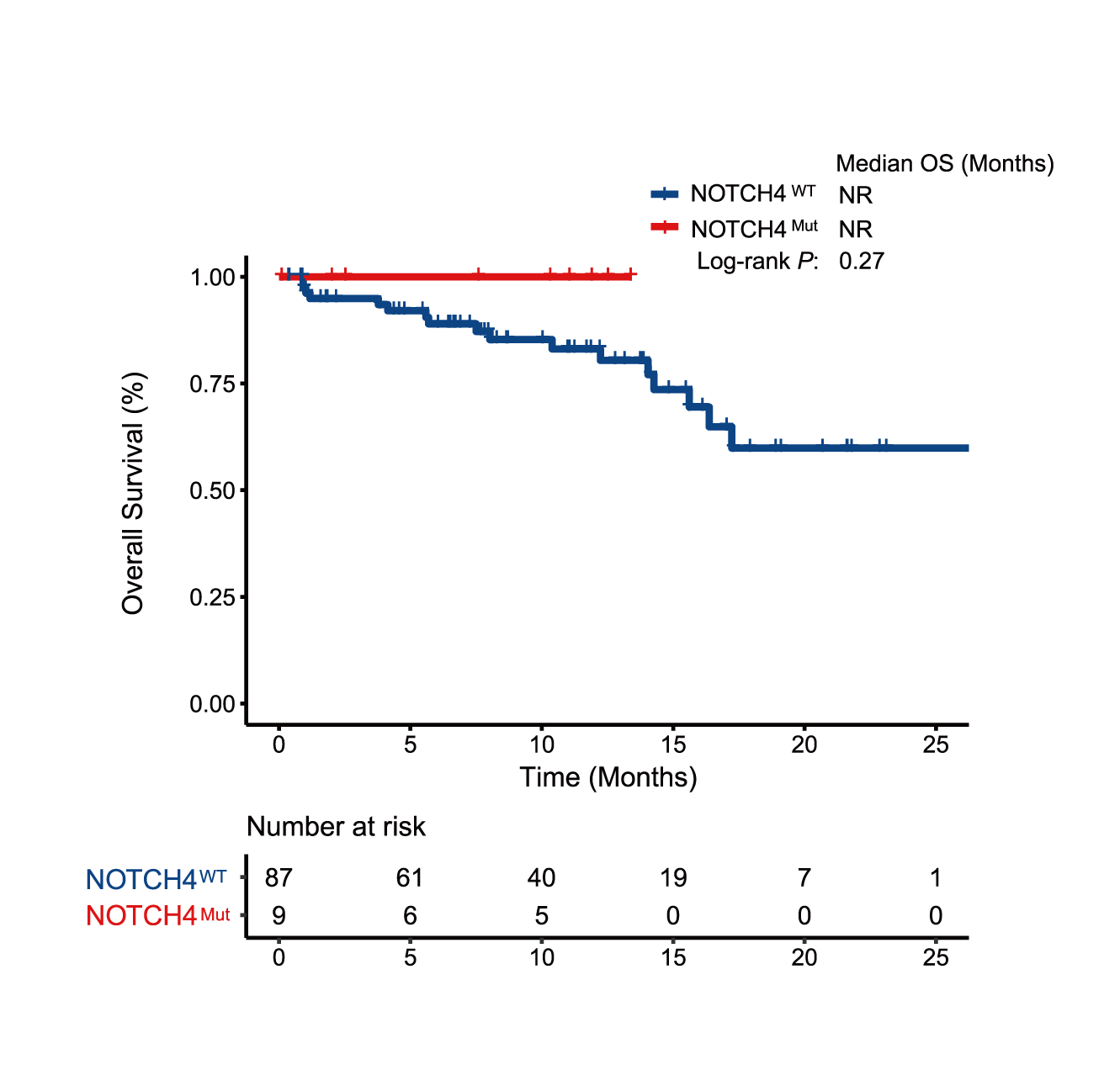

Supplement: Supplementary Figure 2 — The Kaplan-Meier curves of OS between NOTCH4-Mut and NOTCH4-Wt group in the non-ICIs cohort from Zehir cohort. [file Image_2.tif]

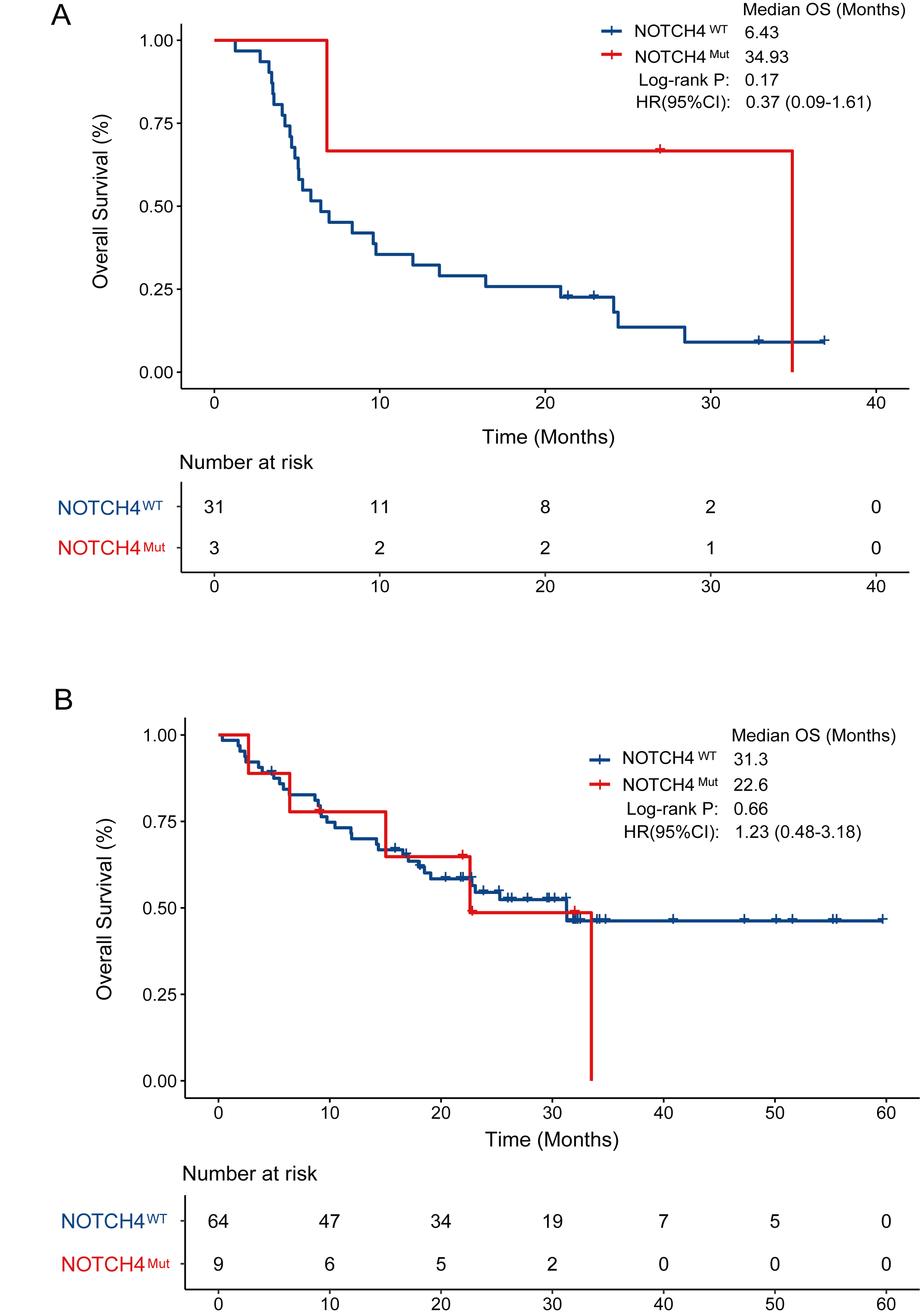

Supplement: Supplementary Figure 3 — The Kaplan-Meier curves of OS between NOTCH4-Mut and NOTCH4-Wt group in NRAS mutant melanoma of discovery cohort (A) and validation cohort (B). [file Image_3.tif]
