# Supplementary material for: NOTCH4 mutation as predictive biomarker for immunotherapy benefits in NRAS wildtype melanoma
Source: Front Immunol. 2022 Jul 29;13:894110. doi: 10.3389/fimmu.2022.894110 (PMC9372281; doi:10.3389/fimmu.2022.894110)
Supplement: Supplementary file 5 [file Table_1.docx]

**Supplementary Table S1** Gene list of DNA damage response gene set used for comparison analysis (from MsigDB)

| **Pathway** | **PathwayID** | **Collection** | **Gene list** |
| --- | --- | --- | --- |
| BER (Base Excision Repair) | R-HSA-73884 | REACTOME | CCNO,POLD3,FEN1,SMUG1,APEX1,LIG1,LIG3,MPG,MUTYH,NTHL1,OGG1,PCNA,POLB,POLD1,POLD2,POLD4,TDG,XRCC1,MBD4 |
| DSB (Double Strand Break Repair) | R-HSA-5696398 | REACTOME | RAD50,XRCC6,H2AFX,LOC389901,LIG1,LIG4,MRE11A,NBN,ATM,TDP1,PRKDC,RAD51,RAD52,RPA1,RPA2,RPA3,LOC651610,BRCA1,BRCA2,TP53BP1,XRCC4,XRCC5,BRIP1,MDC1 |
| FA (Fanconi Anemia) | R-HSA-6783310 | REACTOME | FANCA,FANCC,FANCD2,FANCE,FANCB,FANCF,FANCG,ZBTB32,UBE2T,ATM,ATR,FANCL,FANCM,RPS27A,LOC648152,LOC651610,LOC651921,BRCA1,BRCA2,RPS27AP11,UBA52,RPS27AP11,USP1,PALB2,C17orf70,C19orf40 |
| NER (Nucleotide Excision Repair) | R-HSA-5696398 | REACTOME | CDK7,POLD3,ERCC8,DDB1,DDB2,ERCC1,ERCC2,ERCC3,ERCC4,ERCC5,ERCC6,GTF2H1,GTF2H2,GTF2H3,GTF2H4,LIG1,MNAT1,PCNA,POLD1,POLD2,POLE,POLE2,POLR2A,POLR2B,POLR2C,POLR2D,POLR2E,POLR2F,POLR2G,POLR2H,POLR2I,POLR2J,POLR2K,POLR2L,XAB2,POLD4,RAD23B,RFC2,RFC3,RFC4,RFC5,RPA1,RPA2,RPA3,LOC652672,LOC652857,GTF2H2B,TCEA1,XPA,XPC,CCNH |
| HR (Homologous Recombination) | hsa03440 | KEGG | RAD50,H2AFX,LIG1,MRE11A,NBN,ATM,RAD51,RAD52,RPA1,RPA2,RPA3,LOC651610,BRCA1,BRCA2,TP53BP1,BRIP1,MDC1,RAD50,POLD3,EME1,RAD54B,RPA4,MRE11A,NBN,POLD1,POLD2,POLD4,RAD51,RAD51C,RAD51B,RAD51D,RAD52,RPA1,RPA2,RPA3,BLM,SSBP1,BRCA2,TOP3A,XRCC2,XRCC3,SHFM1,MUS81,RAD54L,TOP3B |
| MMR (Mismatch Repair) | hsa03430 | KEGG | POLD3,MLH3,MSH6,RPA4,LIG1,MLH1,MSH2,MSH3,PCNA,PMS2,POLD1,POLD2,POLD4,RFC1,RFC2,RFC3,RFC4,RFC5,RPA1,RPA2,RPA3,SSBP1,EXO1 |
| NHEJ (NonHomologous End Joining) | hsa03450 | KEGG | RAD50,DNTT,FEN1,XRCC6,POLL,POLM,LIG4,MRE11A,PRKDC,DCLRE1C,LOC731751,XRCC4,XRCC5,NHEJ1 |
| SSB (Single Stranded DNA Binding) | GO:0003697 | Gene Ontology | ERCC1,ERCC4,ERCC5,FUBP1,HMGB2,HNRNPA1,HNRNPA2B1,HNRPDL,IGHMBP2,MLH1,MSH2,MSH3,MYT2,PCBP1,PMS2,POT1,PURA,PURB,RAD23A,RAD23B,RAD51,RAD51AP1,RBMS1,RPA1,RPA2,RPA3,RPA4,SUB1,TERF2,TERF2IP,TP53,TREX1,WBP11,XPC,YBX1 |
| DDR (merged) | na | na | CCNO,POLD3,FEN1,SMUG1,APEX1,LIG1,LIG3,MPG,MUTYH,NTHL1,OGG1,PCNA,POLB,POLD1,POLD2,POLD4,TDG,XRCC1,MBD4,RAD50,XRCC6,H2AFX,LOC389901,LIG4,MRE11A,NBN,ATM,TDP1,PRKDC,RAD51,RAD52,RPA1,RPA2,RPA3,LOC651610,BRCA1,BRCA2,TP53BP1,XRCC4,XRCC5,BRIP1,MDC1,FANCA,FANCC,FANCD2,FANCE,FANCB,FANCF,FANCG,ZBTB32,UBE2T,ATR,FANCL,FANCM,RPS27A,LOC648152,LOC651921,RPS27AP11,UBA52,USP1,PALB2,C17orf70,C19orf40,EME1,RAD54B,RPA4,RAD51C,RAD51B,RAD51D,BLM,SSBP1,TOP3A,XRCC2,XRCC3,SHFM1,MUS81,RAD54L,TOP3B,MLH3,MSH6,MLH1,MSH2,MSH3,PMS2,RFC1,RFC2,RFC3,RFC4,RFC5,EXO1,CDK7,ERCC8,DDB1,DDB2,ERCC1,ERCC2,ERCC3,ERCC4,ERCC5,ERCC6,GTF2H1,GTF2H2,GTF2H3,GTF2H4,MNAT1,POLE,POLE2,POLR2A,POLR2B,POLR2C,POLR2D,POLR2E,POLR2F,POLR2G,POLR2H,POLR2I,POLR2J,POLR2K,POLR2L,XAB2,RAD23B,LOC652672,LOC652857,GTF2H2B,TCEA1,XPA,XPC,CCNH,DNTT,POLL,POLM,DCLRE1C,LOC731751,NHEJ1,FUBP1,HMGB2,HNRNPA1,HNRNPA2B1,HNRPDL,IGHMBP2,MYT2,PCBP1,POT1,PURA,PURB,RAD23A,RAD51AP1,RBMS1,SUB1,TERF2,TERF2IP,TP53,TREX1,WBP11,YBX1 |
