# Supplementary material for: NOTCH4 mutation as predictive biomarker for immunotherapy benefits in NRAS wildtype melanoma
Source: Front Immunol. 2022 Jul 29;13:894110. doi: 10.3389/fimmu.2022.894110 (PMC9372281; doi:10.3389/fimmu.2022.894110)
Supplement: Supplementary file 6 [file Table_2.docx]

**Supplementary Table S2** Detailed clinical information of discovery and validation cohort

| Patient Characteristics | Discovery cohort |  | Validation cohort | | | |
| --- | --- | --- | --- | --- | --- | --- |
|  | Allen 2015 |  | Synder 2014 | Roh 2017 | Riaz 2017 | Liu 2019 |
| Total | 76 |  | 25 | 14 | 53 | 97 |
| Sex |  |  |  |  |  |  |
| Female | 24(32) |  | 7(28) | 5(36) | 0(0) | 41(42) |
| Male | 52(68) |  | 18(72) | 9(64) | 0(0) | 56(58) |
| unknown | 0(0) |  | 0(0) | 0(0) | 53(100) | 0(0) |
| Age,y |  |  |  |  |  |  |
| Mean (SD) | 56.6(18) |  | 58.04(15) | 59.86(13) | NA | NA |
| <65 | 41(54) |  | 14(56) | 8(57) | 0(0) | 0(0) |
| ≥65 | 35(46) |  | 11(44) | 6(43) | 0(0) | 0(0) |
| unknown | 0(0) |  | 0(0) | 0(0) | 53(100) | 97(100) |
| Stage |  |  |  |  |  |  |
| IIIC | 0(0) |  | 0(0) | 0(0) | 0(0) | 6(6) |
| M0 | 8(11) |  | 1(4) | 0(0) | 1(2) | 0(0) |
| M1a | 3(4) |  | 4(16) | 0(0) | 8(15) | 6(6) |
| M1b | 10(13) |  | 3(12) | 0(0) | 7(13) | 12(12) |
| M1c | 55(72) |  | 17(68) | 0(0) | 29(55) | 73(75) |
| unknown | 0(0) |  | 0(0) | 14(100) | 8(15) | 0(0) |
| Best overall response |  |  |  |  |  |  |
| CR | 2(3) |  | 0(0) | 0(0) | 3(6) | 11(11) |
| PR | 10(13) |  | 0(0) | 5(36) | 10(19) | 25(26) |
| SD | 9(12) |  | 0(0) | 1(7) | 15(28) | 14(14) |
| PD | 50(66) |  | 0(0) | 8(57) | 23(43) | 44(45) |
| unknown | 5(6) |  | 25(100) | 0(0) | 2(4) | 3(3) |
| Primary |  |  |  |  |  |  |
| Skin | 63(83) |  | 16(64) | 0(0) | 31(58) | 68(70) |
| Occult | 10(13) |  | 0(0) | 0(0) | 0(0) | 13(14) |
| Mucosal | 3(4) |  | 0(0) | 0(0) | 5(9) | 8(8) |
| Acral | 0(0) |  | 2(8) | 0(0) | 2(4) | 8(8) |
| Ocular | 0(0) |  | 0(0) | 0(0) | 4(8) | 0(0) |
| unknown | 0(0) |  | 7(28) | 14(100) | 11(21) | 0(0) |
| TMB |  |  |  |  |  |  |
| Mean (SD) | 13.7(26) |  | 18.06(22) | 26.23(32) | 11.52(28) | 17.62(38) |
| Drug class |  |  |  |  |  |  |
| CTLA-4 | 76(100) |  | 25(100) | 0(0) | 0(0) | 0(0) |
| PD-1 | 0(0) |  | 0(0) | 0(0) | 53(100) | 97(100) |
| Sequential | 0(0) |  | 0(0) | 14(100) | 0(0) | 0(0) |
| NOTCH4 status |  |  |  |  |  |  |
| Mut | 10(13) |  | 6(24) | 3(21) | 3(6) | 10(10) |
| WT | 66(87) |  | 19(76) | 11(79) | 50(94) | 87(90) |
